# Neutralizing antibody responses assessment after vaccination in people living with HIV using a surrogate neutralization assay

**DOI:** 10.1186/s12865-024-00625-z

**Published:** 2024-07-10

**Authors:** Armel Landry Batchi-Bouyou, Jean Claude Djontu, Line Lobaloba Ingoba, Jiré Séphora Mougany, Freisnel Hermeland Mouzinga, Jacques Dollon Mbama Ntabi, Franck Yannis Kouikani, Arcel Christ Massamba Ndala, Steve Diafouka-kietela, Raoul Ampa, Francine Ntoumi

**Affiliations:** 1 Fondation Congolaise pour la Recherche Médicale (FCRM), Villa D6, Campus OMS, Djoué, Brazzaville, Republic of the Congo; 2 Faculty of Sciences and Techniques, University Marien Ngouabi, Brazzaville, Republic of the Congo; 3 Global Clinical Scholars Research Training Program, Harvard Medical School, Boston, MA USA; 4 Department of Medicine, School of Medicine, Washington University in St Louis, St Louis, MO 63130 USA; 5 Department of Health and Social Care, Ministry of Higher Education, Scientific Research and Technological Innovation, Brazzaville, Republic of the Congo; 6 Ambulatory Treatment Center, National HIV Program, Ministry of Health and Population, Brazzaville, Republic of the Congo; 7 Institute of Tropical Medicine, University of Tübingen, Tübingen, Germany

**Keywords:** Vaccine, Sinopharm/BBIP-CorV, Janssen/Ad26.COV2.S, SARS-CoV-2, Antibodies, Republic of the Congo

## Abstract

**Objective:**

HIV has been reported to interfere with protective vaccination against multiple pathogens, usually through the decreased effectiveness of the antibody responses. We aimed to assess neutralizing antibody responses induced by COVID-19 vaccination in PLWH in Brazzaville, Republique of the Congo.

**Method:**

The study was conducted at the Ambulatory Treatment Center of the National HIV Program, in charge of over 6000 PLWH, and the health center of FCRM in Brazzaville, Republic of the Congo. Participants were divided into two groups: PLWH with well-controlled HIV infection (CD4 counts no older than one week ≥ 800 / mm^3^, undetectable viral load of a period no older than one week and regularly taking Highly Active Antiretroviral Therapy for at least 6 months) and PLWOH. These groups were subdivided by vaccination status: fully vaccinated with adenovirus-based vaccines (Janssen/Ad26.COV2.S and Sputnik/Gam-COVID-Vac) or inactivated virus vaccine (Sinopharm/BBIP-CorV) and a control group of unvaccinated healthy individuals. All participants were RT-PCR negative at inclusion and/or with no documented history of SARS-CoV-2 infection. ELISA method was used for detecting IgG and neutralizing Antibodies against SARS-CoV-2 antigens using a commercial neutralizing assay.

**Results:**

We collected oropharyngeal and blood samples from 1016 participants including 684 PLWH and 332 PLWOH. Both PLWH and PLWOH elicited high levels of antibody responses after complete vaccination with inactivated virus vaccine (Sinopharm/BBIP-CorV) and adenovirus-based vaccines (Janssen/Ad26.COV2.S and Sputnik/Gam-COVID-Vac). Overall, no difference was observed in neutralization capacity between PLWOH and PLWH with well-controlled HIV infection.

**Conclusion:**

The results from this study underline the importance of implementing integrated health systems that provide PLWH the opportunity to benefit HIV prevention and care, at the same time while monitoring their vaccine-induced antibody kinetics for appropriate booster schedules.

**Supplementary Information:**

The online version contains supplementary material available at 10.1186/s12865-024-00625-z.

## Introduction

The original strain of SARS-CoV-2, the causative agent of the COVID-19 pandemic was first identified, sequenced, and made known to the public by December 2019 [1, 2]. Since then, several mutations occurred in the spike protein of the virus which now appears to be more infectious and continues to pose a grave threat to global public health [3–5]. In the absence of efficient treatment, mass vaccination campaigns have been considered a powerful tool to control the pandemic [6–9]. Despite innumerable deaths that have been prevented by vaccines, the gradual effort of governments and WHO to guarantee an equitable distribution of vaccines is being challenged by vaccine hesitancy [10]. High coverage in COVID-19 vaccination is required to mitigate transmission [11], especially in fragile groups such as people living with HIV (PLWH) [10, 12].

Although vaccine hesitancy is multifactorial among PLWH, one major factor is the lack of complete data on their effectiveness including their immunogenicity in an immunocompromised condition. In fact, previous studies have already reported that HIV interferes with protective vaccination against multiple pathogens, usually through the decreased effectiveness of the antibody response [13–17]. In the case of COVID-19 vaccines, neutralizing antibodies are induced after vaccination to confer some protection from potential infection or reinfections [18]. However, data on COVID-19 vaccine effectiveness in PLWH is poorly documented in part because some clinical trials have included relatively few numbers of PLWH, and many authors excluded this group of the population from their trials [19–23]. Likewise, real-world data on antibody responses in PLWH after a COVID-19 vaccine are also limited in most studies which also have been conducted mainly in Europe, America, or Asia with different social determinants of health such as ethnicity found in Africa [24–26]. Today, more than 38 million people worldwide are living with HIV among which approximately 25 million are in sub-Saharan Africa [27]. This is alarming since recent studies still suggest that COVID-19 disease transmissibility, severity, and mortality risks are increased in PLWH [28–31]. In such context, despite laudable efforts of the government to reach a higher vaccine coverage, some concerns about the time to seroconversion post-vaccination, the persistence of neutralizing antibody responses, and eventual sociodemographic factors influencing vaccine-induced antibody kinetic in PLWH are still unclear, making it difficult for healthcare authorities to calibrate vaccination campaigns and design effective booster strategies for this particularly vulnerable group.

Out of the estimated tens of millions of people living with HIV globally, around two-thirds of the projected 38.4 million cases worldwide are concentrated in Africa, highlighting the region’s disproportionate burden of the HIV epidemic [32]. The HIV prevalence in the Republic of the Congo is 3.3% making it the 2nd highest rate in Central Africa and the 14th in the world (UNAIDS data 2021) with an estimated COVID-19 vaccine coverage as low as 11.6% in the general population after 2 years of vaccination campaigns [33–35]. Of the few studies on vaccine effectiveness conducted in Africa [32, 36], one has been conducted in Central Africa precisely in the Republic of the Congo and PLWOH population [37]. Plans for the COVID-19 vaccination boosters should therefore be based on more inclusive scientific data that show substantial and sustained increases and waning in antibody responses on PLWH to reduce vaccine misinformation among this fragile group. The present study aimed to characterize neutralizing antibody responses induced by vaccination in Congolese PLWH by comparing three of the most distributed vaccines in Africa including Sinopharm/BBIP-CorV, Janssen/Ad26.COV2.S, and Sputnik/Gam-COVID-Vac.

## Methods

### Study design, participants, and ethics

The study was conducted from January to September 2022. The sample population was made of two main groups including PLWH and PLWOH.

The PLWH group was made up of HIV-positive volunteers who were regularly followed up at the Ambulatory Treatment Center of the National HIV Program, in charge of about 6000 PLWH in Brazzaville (capital of the Republic of the Congo). Data on the viral loads and CD4 counts of the participants involved in the current study were extracted from the National HIV Program database. Participants of this group were physically healthy HIV-positive individuals with T CD4 counts (no older than one week), of at least 800 / mm^3^, undetectable viral load of a period no older than one week and regularly taking Highly Active Antiretroviral Therapy (HAART) for at least 6 months.

The PLWOH group was made up of healthy volunteers who tested HIV-negative and enrolled at the health center of the Fondation Congolaise pour la Recherche in Brazzaville. These included travelers and healthy volunteers who came for a routine health checkup and consented to participate in the study. As much as possible, enrolled PLWH patients were matched with PLWOH participants according to age, sex, and location.

Demographic characteristics of participants including sex and age were collected in a questionnaire and blood samples and swabs were collected from each volunteer.

These two groups of PLWH and PLWOH were subdivided into two subgroups of fully vaccinated and unvaccinated as follows: (i) Fully Vaccinated with Sinopharm/BBIP-CorV (2 doses), Janssen/Ad26.COV2.S (1 dose) or Sputnik/Gam-COVID-Vac (2 doses) were included at least 2 months after a voluntary vaccination. To be included in this group, participants had to provide an official COVID-19 vaccine certificate delivered by healthcare authorities showing the type of vaccine, date of vaccination, age, and sex. Characteristics of all vaccines investigated during the study are listed in Supplemental Tables 1 [3838–40]. (ii) Control group of unvaccinated healthy individuals. These were vaccine-hesitant volunteers who came for a routine health checkup and consented to participate in the study. At inclusion, all participants who were RT-PCR positive at inclusion and/or reported a documented history of SARS-CoV-2 infection were excluded from the study as well as those under immunosuppressive treatment, those with inflammatory diseases, cancer, cardiovascular diseases, and endocrine and metabolic disorders.

### SARS-CoV-2 detection

RNA was extracted from swabs by the QIAamp Viral RNA Mini Kit (Qiagen, Hilden, Germany) according to instructions and subjected to RealStar® SARS-CoV-2 real-time PCR targeting the S gene of SARS-CoV-2 (Altona Diagnostics, Hamburg, Germany), using a high-performance, high-throughput PCR platform (96 well plates) LightCycler® 480 Instrument II (Roche Diagnostics, Mannheim, Germany).

### SARS-CoV-2 specific antibodies detection

#### Measurement of plasma IgG ab

SARS-CoV-2 anti-S IgG Ab was measured using GSD NovaLisa® SARSCoV-2 (COVID-19) quantitative IgG (NovaTec Immundiagnostica GmbH) according to the manufacturer’s protocol. Briefly, diluted plasma (1:101) and manufacturer-provided controls were incubated in corresponding wells of microplates coated with SARS-CoV-2 antigens for 30 min at 37° C. After washing, each well was incubated with 100 µL of secondary Ab (peroxidase-conjugate F(Ab’)2 fragment, goat anti-human IgG Fc fragment specific), for 30 min at 37° C following by another step of washing. Finally, each well was incubated with 100 µL of enzyme-substrate, tetramethyl benzidine (TMB), and 50 µL of stop solution was added to the different well. The optical density (OD) for each well was immediately measured at 450 nm using an ELISA microplate reader. Quantitative results obtained in Arbitrary Unit/ml (AU/ml) was calculated for each plasma as [OD(from individual plasma)/OD (calibrator Control x correction factor) x 10. These results were converted to International Units (IU/ml) by multiplying 4.5 according to WHO specifications. A sample was considered positive if the ratio was above 49.5 IU/ml. Negative Control, Positive Control, and Calibrator Control (mix of Positive Control with Negative Control) were included in each assay for quality control.

### Measurement of anti-SARS-CoV-2 neutralizing antibodies

A standardized commercial surrogate virus neutralization test (sVNT) was used to assess the neutralizing capacity of plasma Ab. In fact, the cPass™ SARS-CoV-2 Neutralization Antibody Detection Kit (Nanjing GenScript Biotech, China) was used to detect neutralizing antibodies according to the manufacturer’s protocol [41]. The assay strikes a balance between targeting unmutated RBD-conserved regions for broad reactivity while also accounting for some of the more common RBD mutations seen in circulating Variant Of Concern and their sublineages including, Omicron, Delta, Alpha and Beta. After dilution, plasma samples and manufacturer-provided controls were preincubated with peroxidase-conjugated Spike protein receptor-binding domain (HRP-RBD and 100 µL each mixture was then added to the corresponding wells of the capture microplate pre-coated with the human receptor of angiotensin 2 converting enzyme (hACE2) protein and incubated at 37° C for 15 min. Following a wash cycle, 100 µL of enzyme-substrate, tetramethyl benzidine (TMB) was added to each well and the microplate was incubated in dark at 25° C for 15 min. Finally, 50µL of stop solution was added to each well, and the absorbance of the final solution was immediately measured at 450 nm using an ELISA microplate reader. Both negative and positive controls were included in each assay for quality control. A sample was positive for neutralizing antibodies at the manufacturer-recommended of at least 30% of inhibition.

### Statistical analysis

The data were analyzed using SPSS version 24 (SPSS Inc., Chicago, IL, USA). GraphPad (version 8.0.4) was used to generate the figures. Categorical variables were presented as proportions (%). Continuous variables were expressed as median (interquartile range, IQR) or mean (± standard deviation, SD). Mann-Whitney U-test or Kruskal-Wallis test was used to investigate the difference in distributions between two or more groups. For the comparison of categorical variables, Chi-square or Fisher’s exact test was used. Statistical significance was defined as P values of < 0.05. Spearman rank correlation test was used to assess the relationship between antibody level or activity and participant age or post-vaccination period. The sample size was estimated based on the formula adapted to the case-control study,

$$n=\frac{\left(r+1\right)}{r}\frac{\overline{\left(p\right)}\left(1-\overline{p}\right){({Z}_{\beta }+{Z}_{\frac{\alpha }{2}})}^{2}}{{(p1-p2)}^{2}}.$$

Based on 90% expected vaccine-induced seroprevalence in the control group [37] and 84% vaccine-induced seroprevalence in the cases [42] with a threshold of α level of significance set at 0.05, a minimum of 523 cases was required to achieve a statistical power of 80%. The control group represented the pool of participants from whence the cases were drawn [43].

## Results

**Fig. 1:**
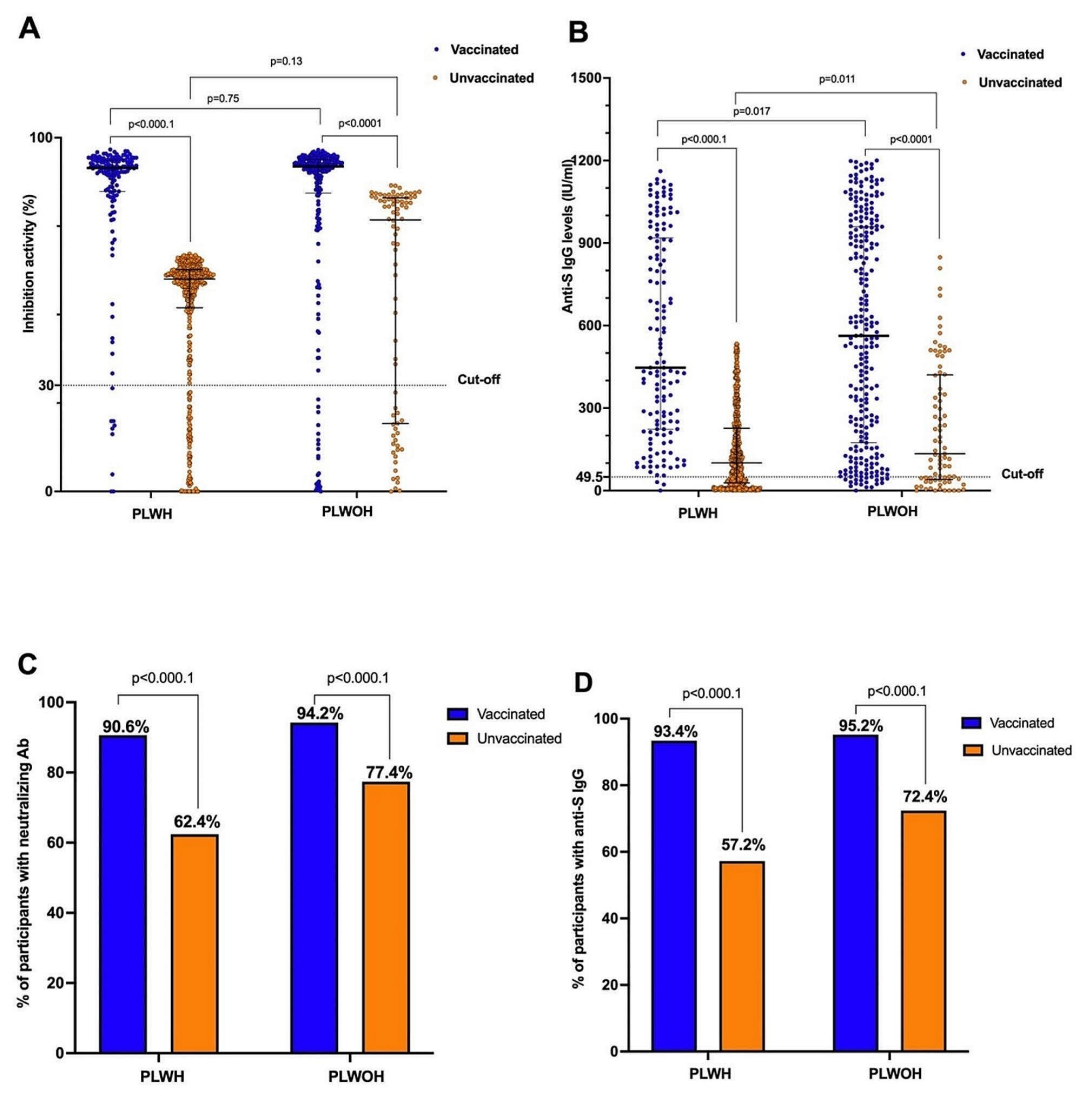
Assessment of antibody responses in vaccinated and unvaccinated participants. Graph **A** shows the inhibition activity of neutralizing antibodies. Anti-S IgG concentrations are represented in graph **B**. Graph **C** shows the proportions of people with neutralizing antibodies and graph **D** represents the proportions of participants with anti-S IgG antibodies. Mann–Whitney Rank Sum test was used for the comparison of antibodies levels and inhibition activity, Fisher exact test for the comparison of the percentage of participants with anti-S IgG and neutralizing antibodies between two groups

A total of 1016 participants were recruited including 684 PLWH and 332 PLWOH. The demographic characteristics of the population are shown in Supplemental Table 2. Overall, plasma from vaccinated PLWH (*n* = 143/684) and vaccinated PLWOH (*n* = 251/332) elicited significantly higher IgG concentrations and inhibition activity than unvaccinated individuals in both groups of PLWH and PLWOH groups (*p* < 0.0001). In addition, over 90% of individuals elicited anti-S IgG and post-vaccination neutralizing antibodies in both groups of PLWH and PLWOH **(**Fig. 1**)**.

### Neutralizing capacity in PLWH and PLWOH according to time post-vaccination

We investigated inhibition activity induced by vaccination over time. Regarding the HIV status, PLWH and PLWOH elicited high levels of inhibition activity after vaccination from 2 to 6 months and 6–12 months (Table 1). When comparing each vaccine, one against the other, the results indicate that Janssen/Ad26.COV2.S (adenovector-based vaccine) induced significantly higher inhibition activity than Sputnik/Gam-COVID-Vac (adenovector-based vaccine), and Sinopharm/BBIP-CorV (inactivated virus vaccine) from 2 to 6 months post-vaccination period in both groups of PLWH (*p* = 0.002) and PLWOH (*p* = 0.012). After 6 months from the vaccination, the inhibition activity was significantly higher with Sinopharm/BBIP-CorV (inactivated virus vaccine) than Sputnik/Gam-COVID-Vac and Janssen/Ad26.COV2.S in both groups of PLWH (*p* = 0.003) and PLWOH (*p* = 0.005) (Table 1).

**Table 1:** Comparison of neutralizing capacity in vaccinated PLWH and PLWOH

2–6 months after vaccination				7–12 months after vaccination		
	PLWH Inhibition activity (%) Median (IQR)	PLWOH Inhibition activity (%) Median (IQR)	**P-value**	PLWH Inhibition activity (%) Median (IQR)	PLWOH Inhibition activity (%) Median (IQR)	**P-Value**
Sinopharm/BBIP-CorV	90.6[78.7 ; 94.2]	92.2[75.5 ; 93.2]	0.21	86.6[60.1 ; 90.4]	89.3[65.5 ; 92.2]	0.23
Sputnik/Gam-COVID-Vac	91.3[76.4; 93.4]	92.3[80.9 ; 94.6]	0.09	85.9[70.6 ; 87.8]	87.2[73,8 ; 90.1]	0.14
Janssen/Ad26.COV2.S	93.9[80.7 ; 95.8]	95.2[80.3 ; 96.1]	0.08	80.1 [62.5 ; 84.6]	85.4 [70.1 ; 87.5]	0.31
**P-value**	**0.002**	**0.012**		**0.013**	**0.022**	

IQR: interquartile range

### Anti-S IgG levels in PLWH and PLWOH according to the post-vaccination period

Overall, both groups of PLWH and PLWOH who were vaccinated elicited higher levels of anti-S IgG than those unvaccinated (*p* < 0.0001) (Table 2). Additionally, vaccination induced a significantly higher concentration of anti-S IgG in PLWOH than in PLWH (*p* = 0.017) (Table 2).

**Table 2:** Comparison of anti-S IgG levels in vaccinated PLWH and PLWOH

2–6 months after vaccination				7–12 months after vaccination		
	PLWH Median IgG concentration (UI/ml) (IQR)	PLWOH Median IgG concentration (UI/ml) (IQR)	**P-value**	PLWH Median IgG concentration (UI/ml) (IQR)	PLWOH Median IgG concentration (UI/ml) (IQR)	**P-Value**
Sinopharm/BBIP-CorV	234.5[164.2 ; 326.7]	376.3[186.2 ; 792.1]	**0.014**	186.4[61.6 ; 199.3]	200.7[103.7 ; 222.4]	**0.031**
Sputnik/Gam-COVID-Vac	412.3[86.4; 93.4]	602.1[452.2 ; 820.7]	**0.001**	155.1[55.3 ; 181.3]	176.1[120.2 ; 195.1]	**0.017**
Janssen/Ad26.COV2.S	533.9[474.2 ; 1032.5]	653.2[470.1 ; 1185.2]	**0.022**	102.5 [50.2 ; 111.2]	152.6 [109.3 ; 288.2]	**0.015**
P-value	**0.005**	**0.009**		**0.003**	**0.005**	

IQR: interquartile range

By comparing each vaccine against the others, the findings show that Janssen/Ad26.COV2.S (adenovector-based vaccine) induced significantly higher inhibition activity than Sputnik/Gam-COVID-Vac (adenovector-based vaccine), and Sinopharm/BBIP-CorV (inactivated virus vaccine) from 2 to 6 months after vaccination in both groups of PLWH (*p* = 0.005) and PLWOH (*p* = 0.009). After 6 months from the vaccination, the inhibition activity was significantly higher in Sinopharm/BBIP-CorV (inactivated virus vaccine) than in Sputnik/Gam-COVID-Vac and Janssen/Ad26.COV2.S in both groups of PLWH (*p* = 0.003) and PLWOH (*p* = 0.005) (Table 2).

### Correlation between age and antibody responses in vaccinated PLWH and PLWOH

In both groups of PLWH and PLWOH, there was no significant correlation between age and anti-S IgG concentrations induced by vaccination (Supplemental Fig. 1A-F). In the PLWOH group, there was a negative slight correlation between age and inhibition activity in all vaccines assessed in the study (Supplemental Fig. 2B, D, and F). No correlation was found between age and inhibition activity in PLWH for all vaccines except a slight negative correlation (*r*= -0.39) for Sputnik/Gam-COVID-Vac (Supplemental Fig. 2E).

### Correlation between post-vaccinal period and antibody responses in vaccinated PLWH and PLWOH individuals

In sera of people who received adenovirus-based vaccines, a negative slight correlation was found between anti-S IgG levels and post-vaccination period with Sputnik/Gam-COVID-Vac vaccinees in both groups of PLWH (*r*= -0.26) and PLWOH (*r*= -0.27), whereas in Janssen/Ad26.COV2.S, there was a slight positive correlation between anti-S IgG and t post-vaccination period in PLWOH (*r* = 0.44) versus a negative trend in PLWH (*r*=-0.34) (FIG). No correlation was observed in the inactivated virus vaccine (Sinopharm/BBIP-CorV) (Supplemental Fig. 3).

We next investigated the correlation between inhibition capacity and post-vaccination period. The findings indicate that there was no correlation in PLWH between inhibition capacity and the post-vaccination period regardless of the type of vaccine (Supplemental Fig. 4A, C, and E). In PLWOH, a weak correlation was found for Sinopharm/BBIP-CorV (*r* = 0.26) and Janssen/Ad26.COV2.S (*r* = 0.20) (Supplemental Fig. 4B, D).

## Discussion

The study sought to match and compare 2 specific groups with closely similar immunological features and, in such cases, give insight into whether the average percentage of inhibition of neutralizing antibodies and the total anti-spike IgG concentrations in both groups would significantly vary depending solely on HIV status after exposure to COVID-19 vaccines.

This study conducted in the Republic of the Congo has provided the first real-world data on COVID-19 vaccine-induced antibody responses in people living with HIV in the Central African region. These data will contribute to local health authorities working with communities to mitigate the growing amount, variety, and spread of misinformation on COVID-19 vaccines which have altered how PLWHs trust preventive measures implemented by health authorities in Africa. This study also provides scientific evidence for stakeholders to make more accessible COVID-19 vaccines for PLWH in low-income countries that are already in demand of better vaccine distribution. This can help for the design of integrated health systems, giving PLWH the opportunity to benefit HIV prevention and care and same time monitor their vaccine-induced antibody kinetics for appropriate booster schedules.

Although previous studies have shown that people with immunosuppression caused by HIV, as indicated by a low CD4 count, have weaker responses to COVID-19 vaccines as a result of persisting immune dysfunction, exhaustion, and immune senescence [44], the current study adds to the growing body of evidence that COVID-19 vaccines induce significant antibody responses in PLWH under a regular regimen of antiretroviral therapy (ART) [25, 26, 45, 46]. More importantly, the present data show no difference in antibody responses to COVID-19 vaccinations between PLWOH and PLWH in certain conditions. This might be explained by the fact participants in the vaccinated PLWH group had a well-controlled HIV infection, thus producing a relatively similar humoral response to PLWOH vaccinee [47, 48].

Participants enrolled within the first six months of vaccination elicited significantly higher IgG levels and inhibition capacity with adenovirus-based vaccines (Janssen/Ad26.COV2.S and Sputnik/Gam-COVID-Vac) than with the inactivated virus vaccine (Sinopharm/BBIP-CorV) in both groups of PLWH and PLWOH. This result is consistent with previous studies indicating a weaker performance of inactivated virus vaccines likely due to the inactivation processes with beta-propiolactone which has been described to be deleterious to the Spike protein and induce moderate antibody responses compared to adenovirus-based vaccines [49–51].

We also investigated vaccine-induced antibody levels among participants seven to twelve months after vaccination. Our results show significantly higher IgG levels and neutralizing activities in inactivated virus vaccine (Sinopharm/BBIP-CorV) than in adenovirus-based vaccines (Janssen/Ad26.COV2.S and Sputnik/Gam-COVID-Vac) in both groups of PLWH and PLWOH. Although cohort studies need to be conducted to clarify these results, it is worth mentioning that inactivated vaccination induces efficient memory B-cell responses in a similar mechanism in response to natural SARS-CoV-2 infection [52–54]. This may result in more persistent vaccine-antibody responses in sera of inactivated virus vaccinees.

There was no correlation between the Ab levels and age in both groups of PLWH or PLWOH. A lack of correlation between age and antibody response post-vaccination has also been reported in several studies [55, 56] while others have reported a negative correlation between post-vaccination antibody response and age [57, 58]. Other correlations, however, are conflicting with regard to variations in IgG levels and neutralizing activity in time for each vaccine. Methodological variations in sample size in each group of vaccinated individuals likely contribute to the variability of the correlation analysis conducted. In the current study, age matching, as well as a larger sample size among vaccinee, could bring out more conclusive results.

The results presented in the current study are subject to some limitations. First, the antibodies detected and neutralization activity measures were not specific to emergent variants since the occurrence of heavily mutated SARS-CoV-2 has raised questions on the potential of breakthrough infections in fully vaccinated individuals due to immune escape [59]. Further studies will help provide an in-depth analysis of vaccine-induced antibody responses specific to SARS-CoV-2 by using high-throughput antibody quantification approaches such as plaque reduction neutralization test (PRNT) [60]. Second, although the current study grouped participants by age of vaccination, we did not investigate the durability of antibodies after full vaccination by a longitudinal approach. Our future investigations will establish causality by conducting a cohort study and using a Cox regression model. Third, it is important to mention that antibody responses account for part of the protective effect of vaccination but not for the entire protection, since T-cell responses were not assessed in this study but would be informative for further investigations.

## Conclusion

Both PLWH and PLWOH elicited high levels of antibody responses after complete vaccination (excluding boosted dose) with inactivated virus vaccine (Sinopharm/BBIP-CorV) and adenovirus-based vaccines (Janssen/Ad26.COV2.S and Sputnik/Gam-COVID-Vac). Overall, no difference was observed in IgG levels and neutralization capacity between PLWH and PLWOH as long as they had a well-controlled HIV infection. This underlines the importance of implementing integrated health systems that provide PLWH the opportunity to benefit HIV prevention and care at the same time with the monitoring of their vaccine-induced antibody kinetics for appropriate booster schedules.

### Electronic supplementary material

Below is the link to the electronic supplementary material.

Supplementary Material 1

## Data Availability

The datasets generated and/or analyzed during the current study are not publicly available due to terms and conditions defined by the local Ethical committee but are available from the corresponding author upon reasonable request.

## Declarations

### Patient and public involvement

Patients and/or the public were not involved in the design, or conduct, or reporting, or dissemination plans of this research.
