# Supplementary material for: Neutralizing antibody responses assessment after vaccination in people living with HIV using a surrogate neutralization assay
Source: BMC Immunol. 2024 Jul 10;25:43. doi: 10.1186/s12865-024-00625-z (PMC11234560; doi:10.1186/s12865-024-00625-z)
Supplement: Supplementary file 1 — Supplementary Material 1 [file 12865_2024_625_MOESM1_ESM.docx]

**SUPPLEMENT MATERIAL**

**Neutralizing Antibody Responses Assessment after Vaccination in People Living with HIV**

**Supplemental Table 1. Characteristics of investigated vaccines.**

| **Vaccine name** | **Manufacturer** | **Active component at first dose** | **Active component at second dose** | **Type** |
| --- | --- | --- | --- | --- |
| Janssen/Ad26.COV2.S | Johnson & Johnson, New Jersey, USA | 5×10^10^ viral particles | NA | Adenovirus- vectored |
| Sputnik/Gam-COVID-Vac | Gamaleya National Research Centre for Epidemiology and Microbiology, Moscow, Russia | (1.0±0.5) x 1011 viral particles | (1.0±0.5) x 1011 viral particles | Adenovirus- vectored |
| Sinopharm/BBIP-CorV | Sinopharm, Beijing, China | 6.5 U (4 mg) of inactivated SARS-CoV-2 antigens | 6.5 U (4 mg) of inactivated SARS-CoV-2 antigens | Inactivated virus |

**Supplemental Table 2: Characteristics of the study population**

| **Variables** | **All**  **N= 1016** | PLWH  **N=684** | PLWOH  **N=332** |
| --- | --- | --- | --- |
| **Age in years (median with IQR)** | 43 (24 ; 54) | 48 (35 ; 56) | 28 (2; 41) |
| **Sex, n (%)**  **Female**  **Male** | 639/1016 (62.9)  377/1016 (37.1) | 472/684 (69)  212/684 (31) | 149/332 (44.9)  183/332 (55.1) |
| **Vaccination status** |  |  |  |
| **Vaccinated, n (%)**  Sinopharm/BBIP-CorV  Sputnik/Gam-COVID-Vac  Janssen/Ad26.COV2.S | 394/1016 (38.7)  104/394 (26.3)  93/394 (23.7)  197/394 (50) | 143/684 (20.9)  27/143 (18.8)  49/143 (34.3)  67/143 (46.8) | 251/332 (75.6)  55/251 (21.9)  66/251 (26.3)  130/251 (51.8) |
| **Unvaccinated individuals, n (%)** | 622/1016 (61.3) | 541/684 (79.1) | 81/332 (24.4) |
| **HAART, n (%)**  INSTI-based triple  INSTI-based dual  NNRTI-based triple | 266 (26.1)  215 (21.1)  203 ( 20.0) | 266 (38.9)  215 (31.4)  203 (29.7) | NA  NA  NA |

N: number of participants in the group; n: number of participants in the subgroup; IQR: interquartile range; INSTI: integrase strand transfer inhibitor; NNRTI: nonnucleoside reverse transcriptase inhibitor; HAART: Highly Active Antiretroviral Therapy

**Supplemental Figure 1.** Assessment of IgG levels against SARS-COV2 spike protein according to the age of PLWH and PLWOH. Graphs represent the correlation between age and IgG levels after vaccination with Janssen/Ad26.COV2.S **(A-B),** Sinopharm/BBIP-CorV **(C-D),** and Sputnik/Gam-COVID-Vac vaccinees (**E-F)**. Dots represent individual donors. Spearman’s ranking test was used to calculate r values**.**

**Supplemental Figure 2. Assessment of inhibition activity of neutralizing antibodies in serum samples from vaccinated PLWH and PLWOH**. Graphs represent the correlation between age and inhibition activity after vaccination with Janssen/Ad26.COV2.S **(A-B),** Sinopharm/BBIP-CorV **(C-D),** and Sputnik/Gam-COVID-Vac vaccinees (**E-F)**. Dots represent individual donors. Spearman’s ranking test was used to calculate r values.

**Supplemental Figure 3.** **Evaluation of post COVID-19 vaccination IgG levels over a period of 12 moinths.** Graphs represent the correlation between the post-vaccinal period and IgG levels after vaccination with Janssen/Ad26.COV2.S **(A-B),** Sinopharm/BBIP-CorV **(C-D),** and Sputnik/Gam-COVID-Vac vaccinees (**E-F).** Dots represent individual donors. Spearman’s ranking test was used to calculate r values.

**Supplemental Figure 4.** **Assessment of post-vaccination inhibition capacity of neutralizing antibodies over 12 months period**. Graphs represent the correlation between the post-vaccinal period and inhibition activity after vaccination in Janssen/Ad26.COV2.S **(A-B),** Sinopharm/BBIP-CorV **(C-D),** and Sputnik/Gam-COVID-Vac **(E-F).** Dots represent individual donors. Spearman’s ranking test was used to calculate r values.
